# Comparison of the Filamentous Fungi Library v4.0 MALDI Biotyper Platform vs MSI-2 performance for identifying filamentous fungi from liquid cultures

**DOI:** 10.1128/jcm.01371-24

**Published:** 2025-01-31

**Authors:** María F. Gonzalez-Lara, Carla M. Román-Montes, Paulette Díaz-Lomelí, Winston Hernández-Ceballos, Lizeth Morales-Camilo, Axel Cervantes-Sánchez, Andrea Cordero-Rangel, Jazmín Tejeda-Olán, Alexandro Bonifaz-Trujillo, Alfredo Ponce-de-León, Areli Martínez-Gamboa

**Affiliations:** 1 Clinical Microbiology Laboratory, Infectious Diseases Department, Instituto Nacional de Ciencias Médicas y Nutrición Salvador Zubirán, Tlalpan, Mexico City, Mexico; 2 Plan of Studies in Medicine (PECEM-MD/PhD), Faculty of Medicine, UNAM 7180 , Mexico City, Mexico; 3 Mycology Laboratory, Dermatology Department, Hospital General de México Dr Eduardo Liceaga 61575 , Mexico City, Mexico; University of Utah, Salt Lake City, Utah, USA

**Keywords:** MALDI-TOF MS, filamentous fungi, fungal identification, fungi library

## Abstract

Correct, rapid, and reliable filamentous fungi identification is crucial for timely diagnosis and therapy. We compared the performance of the FFLv4.0 MALDI Biotyper and MSI-2 to identify filamentous fungi from clinical isolates. We analyzed 307 clinical isolates of *Aspergillus* spp.*, Fusarium* spp.*,* and *Mucorales* and compared them to sequencing as the reference standard. The overall identification rates to genus (*Mucorales*), section (*Aspergillus*), and species complex (*Fusarium*) level were 96% (296/307) for FFLv4.0 and 78.5% (241/307) for MSI-2. By each genus, correct species identification was achieved by FFLv4.0 and MSI-2 as follows: 72.4% (165/228) and 55.3% (126/228) for *Aspergillus* species, 17.6% (6/34) and 38.2% (13/34) for *Fusarium* species, and 88.9% (40/45) and 55.5% (25/45) for the *Mucorales*. The rates of non-identification by FFLv4.0 and MSI-2, respectively, were 4% (9/228) and 18% (41/228) for *Aspergillus* spp., 0% and 17.6% (6/34) for *Fusarium* spp. and 4.4% (2/45) and 42.2% (19/45%) for the *Mucorales*. Misidentification rates by FFLv4.0 and MSI-2, respectively, were 16.2% (37/228) and 6.1% (14/228) for *Aspergillus* species, 67.6% (23/34) and 14.7% (5/34) for *Fusarium* spp., and 0% and 2.2% (1/45) for the *Mucorales*. The FFLv4.0 MALDI Biotyper outperformed MSI-2 in identifying filamentous fungi from liquid culture spectra.

## INTRODUCTION

Filamentous fungi are emerging pathogens recognized as a cause of invasive fungal infections and represent a significant cause of morbidity and mortality among immunocompromised patients, particularly those with neutropenia (1). Rapid and reliable identification is crucial for timely diagnosis and antifungal therapy.

Historically, identifying filamentous fungi involves morphological analysis, which takes time for the fungus to display the characteristic sporulation and hyphae formation, as well as the expertise of highly trained mycologists. This classical approach can often result in inaccurate identification because of morphologically similar closely related species (2). Matrix-assisted laser desorption/ionization (MALDI-TOF) mass spectrometry (MS) has revolutionized clinical microbiology, and it is widely used for the rapid identification of bacteria and yeasts, replacing conventional biochemical automatized identification methods (3, 4). The use of MALDI-TOF MS for filamentous fungi has been delayed because of the reduced diversity of fungal spectra in the commercial libraries (5, 6).

In 2017, the Mass Spectrometry Identification (MSI) database (https://msi.happy-dev.fr/) was developed, a free online platform meant for identifying filamentous fungi using the spectrums obtained by MALDI-TOF (Bruker Daltonics Bremen, Germany) and VITEK MS (bioMérieux Clinical Diagnostic).

The upgraded MSI-2 version identified 83% of 600 types of filamentous fungi analyzed to the species level, whereas the Filamentous Fungi v3.0 MALDI Biotyper Library only identified 22% (7). The MALDI Biotyper has recently updated its Filamentous Fungi Library to version 4, which includes 312 new spectra of 72 species and 11 new genera.

We aimed to compare the performance of the FFL v4.0 MALDI Biotyper and MSI-2 to identify filamentous fungi from clinical isolates from a tertiary care center in Mexico.

## MATERIALS AND METHODS

### Isolates

A total of 307 filamentous fungi isolates were extracted from storage at −80°C at the Clinical Microbiology Laboratory of the Instituto Nacional de Ciencias Médicas y Nutrición Salvador Zubirán (INCMNSZ) and the Mycology Department of the Hospital General de México Dr. Eduardo Liceaga (HGM) between October 2014 and May 2022. Among these were 228 *Aspergillus* isolates, 34 *Fusarium* isolates, and 45 *Mucorales* isolates.

### Molecular identification

We amplified and sequenced β-tubulin and calmodulin genes for *Aspergillus* species (section Fumigati through β-tubulin gene only) as a reference standard, *Fusarium* species by the elongation factor 1α gene (EF1α) and the *Mucorales* by the internal transcribed spacer region.

DNA extraction involved subculture in Sabouraud broth at 30°C, rotation for 24–72 hours for subsequent concentration by centrifugation, and washing with sterile water three times. Mechanical cellular lysis was performed using ¼ inch ceramic beads from MP Biomedicals and the Precellys machine (Bertin Technologies SAS, Parc d’activités du Pas du Lac 10 bis, avenue Ampère 78 180 Montigny-le-Bretonneux FRANCE). The DNA was extracted using the EZNA Fungal DNA Mini Kit from Omega Bio-Tek, following the manufacturer’s instructions. The gene amplification data and procedures are described in the Supplementary Material.

The PCR products were analyzed by agarose gel electrophoresis and were purified with a QIAquick PCR Purification Kit (Qiagen, Hilden, Germany) following the manufacturer’s recommendations.

### DNA sequencing

Sanger sequencing was done with the automatized sequencer 3500 (Applied Biosystems Hitachi, San Francisco, USA). The final reaction was made in a final volume of 10 µL, using 1 µL of the primer, 3 µL BigDye terminator (Applied Biosystems), and 100 ng DNA.

### Sequencing analysis

#### β-tubulin gene sequence of *Aspergillus* section Fumigati

Sequences were analyzed, edited, and compared with those found in the base of GenBank (http://www.ncbi.nlm.nih.gov). Correct species level identification was considered with ≥99.6% of identity with a difference of >0.4% with the closer species.

#### β-tubulin and calmodulin gene sequences of *Aspergillus* non-Fumigati section and elongation factor 1α gene sequences of *Fusarium* spp.

The sequences were analyzed and edited and initially, in the case of *Aspergillus* non-Fumigati, were compared with those found in the base of GenBank (http://www.ncbi.nlm.nih.gov). In contrast, in *Fusarium*, they were compared with those found in the *Fusarium* MLST database (https://fusarium.mycobank.org/). Subsequently, to obtain final identification, the EF1α sequences and the concatenation of β-tubulin and calmodulin were used for phylogenetic tree construction as the best evolutive model and a maximum likelihood analysis with MEGAX with a nonparametric bootstrap using 1,000 replicates.

#### Internal transcribed spacer sequences

These sequences were edited and compared with those found at the International Society for Human and Animal Mycology (ISHAM) barcoding database (https://its.mycologylab.org/) and MYCOBANK database (https://www.mycobank.org/). We considered identification at the species level when identity percentages ≥99.6 with a difference of >0.4% with the closest species were found.

### MALDI-TOF identification

Bruker’s recommendation for liquid culture was followed with modifications; in summary, the isolates were extracted from ultra-freezing, subcultured in Sabouraud agar, and incubated at 30°C until sporulation. A subculture in Sabouraud broth was kept in a spin at room temperature until adequate growth (24–48 hours). The fungal mass was recovered and washed three times with HPLC water and added 300 µL of water and 700 µL of 100% ethanol, was mixed in vortex, and centrifuged at 13,000 rpm for 2 minutes; the supernatant was discarded and with the dry pellet was added 30 µL of 70 formic acid and mixed with vortex and remained for 5 minutes at room temperature; subsequently, 50 µL of acetonitrile was added, mixed with vortex, and kept at room temperature for 5 minutes. It was centrifuged at 13,000 rpm for 2 minutes, 1 µL of the supernatant was taken and placed on the MALDI plate, allowed to dry, and 1 µL of the matrix was added.

Flexcontrol 3.4, MALDI-TOF MS Compass Biotyper, and the Filamentous Fungi Library V4.0 (Bruker, Daltonics Bremen, Germany) were used for identification. The best score from triplicates with concordant identification was selected. A score ≥2 was considered a reliable identification to the species level, a score between 1.7 and <2 to the section/species complex/genus level in the case of *Aspergillus*, *Fusarium,* and *Mucorales*, respectively, and a score <1.7 as a non-identification.

The spectra obtained by the MALDI-TOF Biotyper were also submitted to the MSI-2 database. An A index was interpreted as correct identification at the species level, a B index as correct identification at the section/complex/genus level, and a C index as non-identification (https://msi.happy-dev.fr).

Misidentification was defined when the FFLv4.0 score was >2 or an A index by MSI-2, but molecular identification showed a different species.

### Statistical analysis

Descriptive statistical analysis was made using frequencies and proportions to describe identification rates. Additionally, the Chi2 test was used to determine the difference in proportions. A *P* < 0.05 was considered statistically significant.

## RESULTS

The performance comparison of two MALDI-TOF libraries is shown in Table 1. The correct identification rates to genus (*Mucorales*), section (*Aspergillus*), and species complex (*Fusarium*) level of the 307 filamentous fungi analyzed were 96% (296/307) for FFLv4.0 and 78.5% (241/307) for MSI-2. In contrast, the correct identification at the species level was 69% (211/307) and 53% (164/307), respectively. All analyzed species are shown in Table S1.

**TABLE 1:** Performance comparison of two MALDI-TOF libraries (Filamentous Fungi Library v4.0 and MSI-2) for correct identification of *Aspergillus*, *Fusarium,* and *Mucorales^a^^,^^b^^,^^c^*

	FFL v4.0				MSI2			
Section	Correct section/SC/genus level ID (%)	Correct species level ID (%)	Non-ID (%)	MisID at species level (%)	Correct section/SC/genus level ID (%)	Correct species level ID (%)	Non-ID (%)	MisID at species level (%)
Fumigati (125)	120 (96)	107 (86)^*d*^	5 (4)	3 (2)	98 (78)	87 (70)^*d*^	27 (22)	2 (2)
Flavi (38)	36 (95)	32 (84)	2 (5)	0	31 (81.5)	27 (71)	7 (18)	0
Nigri (36)	35 (97)	10 (28)	1 (3)	24 (67)	33 (92)	4 (11)	3 (8)	9 (25)
Terrei (14)	13 (93)	10 (71)	1 (7)	2 (14)	12 (86)	3 (21)	2 (14)	2 (14)
Versicolor (10)	10 (100)	2 (20)	0	7 (70)	7 (70)	3 (30)	3 (30)	1 (10)
Nidulantes (5)	5 (100)	4 (80)	0	1 (20)	5 (100)	2 (40)	0	0
Species complex								
*F. solani* (18)	18 (100)	3 (17)	0	12 (67)	15 (83)	12 (67)	3 (17)	0
*F. oxysporum* (7)	6 (86)	0	0	7 (100)	7 (100)	1 (14)	0	1 (14.5)
*F. fujikuroi* (5)	5 (100)	3 (60)	0	1 (20)	3 (50)	0	2 (40)	1 (20)
*F. dimerum* (3)	3 (100)	0	0	2 (67)	3 (100)	0	0	3 (100)
*F. incarnatum-F. equiseti* (1)	0 1 (100)	0	0	1 (100)	0	0	1 (100)	0
Genus								
*Mucorales* (45)	43 (95.5)	40 (89)	2 (4)	0	26 (58)	25 (55.5)	19 (42)	1 (2)

^*a*^ SC, species complex; MisID, misidentified; non-ID, unidentified species.

^*b*^ The section/SC/genus level ID column contains the total isolates subtracting the unidentified ones.

^*c*^ For FFLv4, a score ≥2 was considered a reliable identification to the species level and a score between 1.7 and < 2 to the section/species complex/genus level in the case of *Aspergillus, Fusarium,* and *Mucorales*, respectively. A score <1.7 is non-identification. For MSI-2, an A index was interpreted as correct identification at the species level, a B index as correct identification at the section/complex/genus level, and a C index as non-identification.

^*d*^ Indicates the only comparison with a *P* value <0.05.

For *Aspergillus*, correct identification at the section level was 96% (219/228) for FFLv4.0 vs 82% (187/228) for MSI-2, 72% at the species level (165/228) by FFLv4.0, and 55% (126/228) by MSI-2. For the Fumigati section identification by FFLv4.0, of 125 isolates, 107 were correct species level ID plus 5 non-identified and 3 misidentified, and 10 correct section level ID; by MSI2, of a total of 125, 87 were correct species level ID, 27 unidentified (no ID), 2 misidentified, and 9 correct section level ID.

The highest rate of correct identification at the species level was seen in the Fumigati section (86%, 107/125) and the Flavi section (84%, 32/38) for FFLv4.0, compared to 70% (87/125) for the Fumigati section and 71% (27/38) for the Flavi section with MSI-2. Both libraries correctly identified *Aspergillus unguis*, *Aspergillus flavus*, and *Aspergillus tamarii*.

For *Fusarium*, correct identification at the species complex level was 97% (33/34) for FFLv4.0 and 82% (28/34) for MSI-2. At the species level, the correct identification was 18% (6/34) and 38% (13/34) by FFLv4.0 and MSI-2, respectively. FFLv4.0 had 100% correct identifications at the species complex (SC) level except for the *F. oxysporum* species complex (FOSC), which only had 86% (6/7). In comparison, MSI-2 achieved correct identification of 100% FOSC and *Fusarium dimerum* species complex (FDSC), while for the *Fusarium solani* species complex (FSSC), *F. fujikuroi* species complex (FFSC), and *F. incarnatum-equiseti* (FIESC), this library did not identify six isolates. The highest rates of correct identification at the species level were obtained for FFSC with 60% by FFLv4.0 and for FSSC with 66% by MSI-2.

For the *Mucorales*, correct identification at the genus level was 95.5% (43/45) for FFLv4.0 and 58% (26/45) for MSI-2. Correct species level identification was achieved in 89% (40/45) by FFLv4.0 and 55.5% (25/45) by MSI-2. The detailed performance comparing FFLv4.0 with MSI-2 is shown in Table 1.

The overall misidentification rate at the species level was 19.5% (60/307) for FFLv4.0 and 6.5% (20/307) for MSI-2. For *Aspergillus*, FFLv4.0 and MSI-2 found 16% (37/228) and 6% (14/228), respectively, and for *Fusarium*, 68% (23/34) and 15% (5/34). FFLv4.0 found no misidentifications for the *Mucorales*, while MSI-2 found 2% (1/45).

Misidentification was more frequent among species in section Nigri with FFLv4.0 (66%, 24/36) and MSI-2 (25%, 9/36) and section Versicolores (70%, 7/10) with FFLv4.0. For *Fusarium*, FFLv4.0 had higher misidentification rates in all complexes, while MSI-2 misidentified only three FDSC isolates, one FFSC, and one FOSC isolate. For the *Mucorales*, MSI-2 incorrectly identified one *Mucor racemosus* as *Mucor plumbeus*. Table 2 shows a detailed description.

**TABLE 2:** Misidentification at species level by FFLv4.0 and MSI-2 comparing with molecular identification (ID)

Molecular ID	ID by FFLv4.0	Molecular ID	ID by MSI-2
*Aspergillus* species		*Aspergillus* species	
Fumigati section		Fumigati section	
*A. fumisynematus*^*a*^ (2)	*A. lentulus*	*A. hiratsukae*^*b*^ (1)	*A. lentulus*
*A. fumigatus* (1)	*A. lentulus*	*A. fumigatus* (1)	*A. fisheri*
Nigri section		Nigri section	
*A. costaricaensis*^*a*^ (1)	*A. niger*	*A. tubingensis*^*b*^ (1)	*A. brasiliensis*
*A. neoniger*^*b*^ (5)	*A. niger*	*A. tubingensis*^*b*^ (1)	*A. niger*
*A. piperis*^*b*^ (1)	*A. niger*	*A. neoniger*^*b*^ (3)	*A. tubingensis*
*A. tubingensis*^*b*^ (9)	*A. niger*	*A. costaricaensis*^*a*^ (1)	*A. tubingensis*
*A. luchuensis*^*b*^ (3)	*A. niger*	*A. luchuensis*^*b*^ (1)	*A. tubingensis*
*A. phoenix*^*a*^ (1)	*A. niger*	*A. niger* (1)	*A. welwitschiae*
*A. welwitschiae*^*b*^ (4)	*A. niger*	*A. phoenix*^*a*^ (1)	*A. welwitschiae*
Terrei section		Terrei section	
*A. neoafricanus*^*a*^ (1)	*A. terreus*	*A. neoafricanus*^*a*^ (1)	*A. alabamensis*
Versicolores section		*A. terreus* (1)	*A. niveus*
*A. sydowii* (Sydowii clade) (6)	*A. versicolors* (Versicolors clade)	Versicolores section	
*A. creber*^*b*^ (Sydowii clade) (1)	*A. versicolors* (Versicolors clade)	*A. sydowii* (Sydowii clade) (1)	*A. amoenus* (Versicolors clade)
Nidulantes section		*F. oxysporum* SC	
*A. quadrilineatus*^*b*^ (1)	*A. nidulans*	*F. oxysporum* SC	*F. nirenbergiae*
*Fusarium* spp.		*F. fujikuroi* SC	
*F. solani* SC		*F. temperatum*^*a*^ (1)	*F. proliferatum*
*F. pseudensiforme*^*a*^ (1)	*F. solani*	*F. dimerum* SC	
*F. petroliphilum* (10)	*F. solani*	*Fusarium* sp. Strain 56.93^*a*^ (2)	*F. dimerum*
*Fusarium* sp. FSSC25^*a*^ (1)	*F. solani*	*Fusarium* probable new species^*a*^ (1)	*F. dimerum*
*F. oxysporum* SC		*Mucorales*	
*F. veterinarium*^*b*^ (2)	*F. oxysporum*	*Mucor racemosus* (1)	*Mucor plumbeus*
*F. nirenbergiae* (3)	*F. oxysporum*		
*F. nirenbergiae* (1)	*F. proliferatum* (FFSC)		
*F. contaminatum*^*a*^ (1)	*F. oxysporum*		
*F. fujikuroi* SC			
*F. temperatum*^*a*^ (1)	*F. proliferatum*		
*F. dimerum* SC			
*Fusarium* sp. Strain 56.93^*a*^ (1)	*F. delphinoides*		
*Fusarium* probable new species^*a*^ (1)	*F. delphinoides*		

^*a*^ Species not available in Filamentous Fungi Library v4.0 or MSI-2.

^*b*^ Species not available in Filamentous Fungi Library v4.0.

Discrepancies between databases were observed in *F. petroliphilum* (FSSC); MSI-2 correctly identified 12/13, but only one by FFLv4.0; the rest were misidentified as *F. solani*. Also, *F. veterinarium* was correctly identified by MSI-2 but misidentified by FFLv4.0 as *F. oxysporum*. Finally, *F. verticillioides* was identified by FFLv4.0 but not by MSI-2.

The overall no-identification rates were 4% (11/307) by FFLv4.0 and 21.5% (66/307) by MSI-2, respectively. They were 4% (9/228) and 18% (41/228) for *Aspergillus* spp., 0% and 18% (6/34) for *Fusarium* spp., and 4% (2/45) and 42% (19/45) for the *Mucorales* by FFLv4.0 and MSI2, respectively. A detailed description is shown in Table 3.

**TABLE 3:** Unidentified isolates by FFLv4.0 and MSI-2

FFLv4.0	MSI-2	FFLv4.0	MSI-2	FFLv4.0	MSI-2
*Aspergillus* species *n*= (%)		*Fusarium* species *n*= (%)		*Mucorales n*= (%)	
Fumigati section *n* = 125		*F. solani* SC *n* = 18			
*A. fumigatus* 3/117 (2.6)	*A. fumigatus* 25/117 (21.4)		*F. solani* 1/3 (33)	*Rhizopus arrhizus* 1/27 (4)	*Rhizopus arrhizus* 11/27 (41)
*A. hiratsukae^a^* 2/3 (67)	*A. hiratsukae^a^* 1/3 (33)		*F. petroliphilum* 1/13 (7.7)	*Mucor racemosus* 1/2 (50)	*Rhizopus microsporus* 1/5 (20)
	*A. lentulus* 1/3 (33)		*Fusarium* sp. FSSC25^*b*^ 1/1 (100)		*Mucor circinelloides* 2/4 (50)
Flavi section *n* = 38		*F. fujikuroi* SC *n* = 7			*Mucor racemosus* 1/2 (50)
*A. flavus* 2/35 (6)	*A. flavus* 6/35 (17)		*F. verticillioides* 1/3 (33)		*Rhizomucor pusillus* 1/3 (33)
	*A. tamari* 1/3 (33)		*F. temperatum^b^*1/1 (100)		*Lichtheimia ramosa* 1/1 (100)
Nigri section *n* = 36		*F. incarnatum*-*equiseti* SC *n* = 1			*Syncephalastrum racemosum* 1/2 (50)
*A. costaricaensis^b^* 1/2 (50)	*A. costaricaensis^b^* 1/2 (50)		*F. equiseti* 1/1 (100)		
	*A. welwithschiae^a^* 1/4 (25)				
Terrei section *n* = 14					
*A. terreus* 1/12 (8)	*A. terreus* 1/12 (8)				
	*A. niveus^a^* 1/1 (100)				
Versicolores section *n* = 10					
	*A. sydowii* 3/8 (37)				

^*a*^ Not available in Filamentous Fungi Library v4.0.

^*b*^ Not available in Filamentous Fungi Library v4.0 or MSI-2.

After excluding the 44 isolates with spectra absent from FFLv4.0 and the 14 isolates absent from MSI-2, the frequency of correct identification remained similar (see online supplementary material 1). However, erroneous identification at the species level for these isolates was observed in 86% of cases for FFLv4.0 and in 64% of cases for MSI-2. This was unexpected, as these isolates would have ideally remained unidentified or been identified at a higher level.

## DISCUSSION

In this study, we compared the performance of two MALDI-TOF libraries (FFLv4.0 and MSI-2) to identify a collection of filamentous fungi previously characterized by sequencing. Correct identification was achieved by FFLv4.0 in 96% considering section level for *Aspergillus*, species complex level for *Fusarium,* and genus level for the *Mucorales*, while MSI-2 achieved 78%. At the species level, correct identification was achieved in 69% with FFLv4.0 and 53% with MSI-2. This is an improvement since FFLv3.0 correctly identified 22% of isolates to the species level in a previous report, although identification was carried out from solid culture (7). Still, 4% and 21.5% were not identified by FFLv4.0 and MSI-2, respectively. A robust library is critical for the adequate identification of filamentous fungi with MALDI-TOF (8). The FFLv3.0 contained spectra for 175 species but was recently updated to its 4th version, containing 247 species and 27 strains identified to the genus level. A total of 73 different genera are included, which may explain the improvement seen in this study (9). However, MSI-2 contains more than 1,300 species, so a better performance was expected if it only depended on the species’ diversity.

To improve the performance of MALDI-TOF identification, the protein extraction procedure must be the same as the one used to create the reference library. Our study extracted the proteins from a liquid culture Bruker uses to generate its FFL. Conversely, MSI-2 used a solid culture protein extraction. Previous studies showed a better performance of MSI-2 compared to FFLv3.0 when protein extraction was made from a solid culture (7, 10). Of note, liquid cultures are infrequently used in clinical laboratories since MALDI-TOF identification is usually made from solid media, which reduces the workload and the time to obtain the results, which can reduce the time to adequate antifungal treatment (11). Bruker has two new methods: tube extraction and direct deposition on the MALDI-TOF plate. The proposal is to conduct rapid extraction methods and analyze the FFL and MSI-2.

Also, due to geographical variations, intrinsic differences may exist between the isolates included in the libraries and those analyzed in each study (12, 13). In the specific case of *Aspergillus*, the MSI-2 library has 80 references for *Aspergillus fumigatus* and 56 for *A. flavus* oryzae group, and even so, it failed to identify 21.4% (25/117) isolates of *A. fumigatus* and 17% (6/38) of *A. flavus* oryzae group. The same happened with 41% (11/27) *Rhizopus arrhizus*, which could not be identified even though MSI-2 contains 40 references for this organism. Unfortunately, there is a lack of information on the origin of the isolates used to construct either library.

Several studies show an improvement in identification with rates over commercial libraries supplemented with in-house isolate spectra (6, 14, 15); however, this may not be practical for diagnostic laboratories since molecular diagnostic capacity, including sequencing and personnel trained in correct spectra generation and editing, is required.

The FFLv4.0 had a lower rate of non-identified isolates; however, it had a higher rate of misidentification at the species level when compared with MSI-2. Species-level misidentifications with FFLv4.0 were frequent, with rates were over 60% for *Aspergillus* section Nigri which can be due to FFLv4.0 only containing spectra for *Aspergillus niger*, *Aspergillus japonicus*, and *Aspergillus brasiliensis*; therefore, the rest of the cryptic species cannot be identified (16). *A. niger sensu stricto* were the only isolates correctly identified to species level. The remaining isolates of other cryptic species (*Aspergillus tubingensis*, *Aspergillus luchuensis*, *Aspergillus welwitschiae*, *Aspergillus neoniger*, *Aspergillus phoenix*, *Aspergillus costaricaensis*, and *Aspergillus piperis*) were misidentified as *A. niger* with scores ≥2. A likely explanation is that they are strongly molecularly related species, and molecular methods and phylogenetic tree construction are required to achieve species identification of section Nigri (17–19). Our results show that FFLv4.0 requires curation to maintain only spectra from molecularly identified isolates. Furthermore, exploring the possibility of identifying these species through protein spectra is a priority. Identification of the species level is essential for epidemiological purposes because cryptic species of the Nigri section may differ significantly in terms of antifungal susceptibility (20). It is worth mentioning that MSI-2 has spectra of several species of the Nigri section; despite that, it only achieved identification at the section level. Regarding section Versicolores, FFLv4.0 only contains spectra of *Aspergillus versicolor* and of *Aspergillus sydowii*; still, six were identified as *A. versicolor*, which was also described for FFLv3.0 (10). This section comprises three clades, clade *A. sydowii*, clade *A. versicolor*, and clade *A. subversicolor* (21). Since these are closely related species, it is necessary to characterize them by sequencing before including them in the libraries to avoid misidentifications.

All *Fusarium* isolates analyzed were correctly identified to a complex level by both libraries, except for one *F. oxysporum* SC by FFLv4.0. However, correct species-level identification rates were poor in both libraries, being the lowest for FFLv4.0 (18%), similar to another report (8). We found 67% misidentifications at the species level within the FSSC by FFLv4.0. This may be related to the underrepresentation of the species in FFLv4.0 or intrinsic differences within isolates used to build FFLv4.0 and those analyzed in this study (8). The same happened with *F. veterinarium*, which was correctly identified by MSI-2 and misidentified by FFLv4.0 (although this library does not contain spectra for this species). In contrast, *F. verticillioides* was identified only by FFLv4.0 despite MSI-2 containing 64 spectra for this species. This may be related to the use of liquid instead of solid cultures. Among the two absent species in the library (*F. pseudensiforme* and *Fusarium* sp. FSSC25), they could be identified at the species complex level. All other misidentifications within *Fusarium* could be identified at the complex level, which may be enough for clinical decision-making. The identification of *Fusarium* species remains a challenge even by molecular methods since databases such as GenBank, FUSARIOID-ID database (www.fusarium.org), or CBS-KNAW (Westerdijk Fungal Biodiversity Institute; https://wi.knaw.nl) cannot always guarantee unambiguous identification (10), again, reinforcing the need for curating and updating MALDI-TOF libraries.

We found improved performance of FFLv4.0, compared to MSI-2, for identifying *Mucorales* at the species level, which agrees with previous reports (8). In contrast to our results, a recent study showed 95% identification of the species level for MSI-2 and 90% for FFLv3.0 for 20 analyzed isolates, although identification was carried out from solid culture (10). In our study, FFLv4.0 identified two *Syncephalestrum racemosus*, while MSI-2 could not identify any. In the case of *Mucorales*, even though studies show differences in mortality rates, virulence, and *in vitro* antifungal activity, there is little evidence that identification to genus or species level can guide the choice of antifungal treatment to be implemented (22–28). Still, the relevance of species-level identification relies on expanding the knowledge in epidemiology and investigating outbreaks.

It is essential to know the performance of the libraries to avoid errors in reporting results even when the FFLv4.0 and MSI-2 give results with a score ≥2 or an A index. Based on our results, the *Mucorales* can be reliably reported at the genus and species level. In contrast, most *Aspergillus* and *Fusarium* should be reported at the section and species complex levels, respectively. However, both libraries correctly identified *A. unguis*, *A. tamari*, and *A. flavus/orizae*. In addition, *F. petroliphilum* and F. *veterinarium* can be reported at the species level by MSI-2 and *F. verticillioides* by FFLv4.0. This means that both libraries are needed and may complement the described species. Liquid culture improves the performance of FFLv4.0; however, this type of culture is not practical for routine clinical laboratories since it requires more prolonged incubation and additional tube extraction with ethanol, formic acid, and acetonitrile. While conventional identification from solid culture shortens the turnaround time, previous studies have shown that it negatively impacts the results obtained by this library. In a study by Barker et al., the performance of FFLv3.0 showed results similar to those of MSI-2 from plate culture, which were similar to our identification rate (10). We considered performing extraction from a liquid culture, which, although not a widely used method in clinical settings, was used by the manufacturer to construct the library. This can be helpful in cases where identification is not successful when extraction is performed from solid culture. As mentioned, a crucial step for successful identification is using the same extraction method to create the library.

The performance of the most recent version, FFLv5.0, by directly performing extraction on the disposable plates and analysis by the new MALDI Biotyper SIRIUS platform (MBT HT Filamentous Fungi IVD) remains to be known.

We recognize some limitations: the isolates were clinical samples from two centers in Mexico City. Also, we did not test solid cultures, which may have limited MSI-2 performance. However, we cannot rule out the possibility that MSI-2 requires expansion of spectra diversity.

The main strength of this study is the large number of isolates identified by sequencing and also the external validation of libraries with isolates from a region not previously reported in other studies. MALDI-TOF has achieved remarkable progress in identifying filamentous fungi in recent years, particularly for those laboratories that still need highly trained personnel in mycology. Work must continue to obtain more robust libraries to identify the main fungal species in the short term. This study aims to narrow the current gap of MALDI-TOF diagnosis for filamentous fungi, which lags the impact on bacterial diagnosis, an area in which progress over the years has been relatively rapid and essential. Also, this is the first reported comparison with the FFLv4.0 to our knowledge.

We conclude that the MALDI Biotyper FFLv4.0 outperformed MSI-2 when analyzing spectra from a liquid medium culture. *Mucorales*, *Fusarium*, and *Aspergillus* spp. were reliably identified regarding genus, species complex, and section level. Keeping commercial and public libraries updated and curated is of the utmost importance to improving the diagnosis of the clinical microbiology laboratory.

## SUPPLEMENTAL MATERIAL

Supplemental material jcm.01371-24-s0001.docx Table S1, analyzed species of filamentous fungi; Text S1, additional data of molecular identification.

ASM does not own the copyrights to Supplemental Material that may be linked to, or accessed through, an article. The authors have granted ASM a non-exclusive, world-wide license to publish the Supplemental Material files. Please contact the corresponding author directly for reuse.
