# Supplementary material for: Comparison of the Filamentous Fungi Library v4.0 MALDI Biotyper Platform vs MSI-2 performance for identifying filamentous fungi from liquid cultures
Source: J Clin Microbiol. 2025 Jan 31;63(3):e01371-24. doi: 10.1128/jcm.01371-24 (PMC11898572; doi:10.1128/jcm.01371-24)
Supplement: Supplemental material — Table S1, analyzed species of filamentous fungi; Text S1, additional data of molecular identification. [file jcm.01371-24-s0001.docx]

**Comparison of the Filamentous Fungi Library v4.0 MALDI Biotyper platform vs. MSI-2 performance for identifying filamentous fungi.**

**Supplementary material**

Table S1. Analyzed species of filamentous fungi.

| ***Aspergillus*** | ***Fusarium*** | ***Mucorales*** |
| --- | --- | --- |
| **Section** | **Complex** | **Genera** |
| **Section Fumigati (125)**  *A. fumigatus*  (117)  *A. lentulus*  (3)  *A. hiratsukae*  (3)  *A. fumisynnematus* (2) | ***F. solani* SC *(18)***  *F. petroliphilum*  (13)  *F. pseu* *densiforme* (1)  *F. solani sensu estricto* (3)  *Fusarium sp. FSSC25*  (1) | ***Mucorales* (45)**  *R. arrhizus* (27)  *R. microsporus* (5)  *M. circinelloides* (4)  *M. racemosus* (2)  *R. pusillus* (3)  *L. ramosa* (1)  *L. corymbifera* (1)  *S. racemosum* (2) |
| **Section Flavi (38)**  *A. flavus* (35)  *A. tamari* (3) | ***F. oxysporum* SC** (7)  *F. veterinarium* (2)  *F. contaminatum* (1)  *F. oxysporum SC* (4) |  |
| **Section Nigri (36)**  *A. niger* (10)  *A. costaricaensis* (2)  *A. luchuensis* (3)  *A. neoniger* (5)  *A. phoenix*  (2)  *A. piperis* (1)  *A. tubingensis*  (9)  *A. welwitschiae* (4) | ***F. fujikuroi SC*** (5)  *F. verticillioides* (3)  *F. proliferatum*  (1)  *F. temperatum*  (1) |  |
| **Section Terri (14)**  *A. terreus*  (12)  *A. niveus* (1)  *A. neoafricanus*  (1) | ***F. dimerum* SC** (3)  *Fusarium sp. 56.93* (2)  *Fusarium* probable new species of FDSC (1) |  |
| **Section Versicolores (10)**  *A*. *sydowii* (8)  *A*. *versicolor*  (1)  *A*. *creber*  (1) | ***F. incarnatum-*** ***equiseti* SC** **(1)**  *F. equiseti*  (1) |  |
| **Section Nidulantes (5)**  *A. quadrilineatus* (1)  *A. nidulans* (2)  *A. unguis* (2) |  |  |

**S1. Molecular identification**

**β-tubulin gene amplification.** The primers used were BT2a 5´-GGTAACCAAATCGGTGCTGCTTTC-3´ and BT2b 5´-ACCCTCAGTGTAGTGACCCTTGGC-3´ (CLSI MM18-A, April 2008). The final mix consisted of a volume of 50 µL, with 1X buffer, 1 Mm MgCl_2_, 100 µM dNTPs (Roche Diagnostics, Germany), 0.5 pmol of each primer, 1U Taq polymerase (Invitrogen, Thermo Fisher, Scientific Baltics UAB, Vilnius, Lithuania) and 100 ng DNA. Cycling conditions were 95°C for 10 min, 35 cycles at 95°C for 30 seconds (sec), 55°C for 45 sec, 72°C for 60 sec, and a final extension at 72°C for 10 min.

**Calmodulin gene amplification for *Aspergillus* section Flavi identification.** The primers used were cmdA7 5´-GCCAAAATCTTCATCCGTAG-3´ and cmdA8 5´-ATTTCGTTCAGAATGCCAGG-3´. The final mix consisted of a volume of 50 µL, with 1X buffer, 1.5 Mm MgCl_2_, 200 µM dNTPs (Roche Diagnostics, Germany), 0.2 pmol of each primer, 0.5 U Taq polymerase (Invitrogen, Thermo Fisher, Scientific Baltics UAB, Vilnius, Lithuania) and 100 ng DNA. Cycling conditions were 94°C for 7 min, 35 cycles at 94°C for 60 sec, 56°C for 60 sec, 72°C for 60 sec, and a final extension at 72°C for 5 min.

**Calmodulin gene amplification for *Aspergillus* section Nigri, Terri, and Versicolor identification.** The primers used were CL1 5´-GAGATATCAAGGAGGCCTTCTC-3´ and CL2A 5´-TTTTTGCATCATGAGTTGGAC-3´. The final mix consisted of a volume of 100 µL, with 1X buffer, 1.5 Mm MgCl_2_, 200 µM dNTPs (Roche Diagnostics, Germany), 0.5 pmol of each primer, 2.5 U Taq polymerase (Invitrogen, Thermo Fisher, Scientific Baltics UAB, Vilnius, Lithuania) and 100 ng DNA. Cycling conditions were 95°C for 10 min, 35 cycles at 94°C for 50 sec, 55°C for 50 sec, 72°C for 60 sec, and a final extension at 72°C for 7 min.

**Elongation factor 1α gene amplification.** The primers used were EF-1 5´-ATGGGTAAGGARGACAAGAC-3´ and EF-2 5´-GGARGTACCAGTSATCATG-3´ as the CLSI MM18-A April 2008 recommended. The final mix consisted of a volume of 25 µL, with 1X buffer, 1 Mm MgCl_2_, 200 µM dNTPs (Roche Diagnostics, Germany), 0.5 pmol of each primer, 1U Taq polymerase (Invitrogen, Thermo Fisher, Scientific Baltics UAB, Vilnius, Lithuania) and 100 ng DNA. Cycling conditions were 95°C for 10 min, 35 cycles at 95°C for 45 sec, 50°C for 45 sec., 72°C for 60 sec, and a final extension at 72°C for 7 min.

**Internal transcribed spacer amplification**. For ITS amplification, we used the primers ITS-5 5´-GGAAGTAAAAGTCGTAACAAGG-3´ e ITS-4 5´-TCCTCCGCTTATTGATATGC-3´ following CLSI MM18A recommendations in April 2008. The final mix consisted of a volume of 50 µL, with 1X buffer, 1.5 Mm MgCl_2_, 100 µM dNTPs (Roche Diagnostics, Germany), 0.5 pmol of each primer, 2.5 U Taq polymerase (Invitrogen, Thermo Fisher, Scientific Baltics UAB, Vilnius, Lithuania) and 100 ng DNA. Cycling conditions were 95°C for 7 min, 35 cycles at 94°C for 60 sec, 50°C for 30 sec, 72°C for 45 sec, and a final extension at 72°C for 7 min.
